# Can Leaves and Stems of *Rubus idaeus* L. Handle *Candida albicans* Biofilms?

**DOI:** 10.3390/ph13120477

**Published:** 2020-12-18

**Authors:** Clément Bernard, Camille Juin, Marine Vitry, Van Thanh Danh Le, Julien Verdon, Anne-Solène Toullec, Christine Imbert, Marion Girardot

**Affiliations:** Laboratoire Ecologie et Biologie des Interactions, Université de Poitiers, UMR CNRS 7267, F-86073 Poitiers, France; clement.bernard@univ-poitiers.fr (C.B.); camillejuin@orange.fr (C.J.); marinevitry4@gmail.com (M.V.); le-danh@hotmail.com (V.T.D.L.); julien.verdon@univ-poitiers.fr (J.V.); toullecannesolene@gmail.com (A.-S.T.); christine.imbert@univ-poitiers.fr (C.I.)

**Keywords:** *Rubus idaeus* L., raspberry, *Rosaceae*, *Candida albicans*, anti-biofilm, bioguided fractionation, stems and leaves

## Abstract

*Candida albicans* is an opportunistic pathogen involved in many infections, especially linked to implanted medical devices. Its ability to form biofilms complicates the treatment of these infections as few molecules are active against sessile *C. albicans*. The aim of this study was to evaluate the potential of leaves, three-month-old and one-year-old stems of *Rubus idaeus* L. against *C. albicans* biofilm growth. Extractions with a polarity gradient were carried out on hydroacetonic extracts and followed by fractionation steps. The obtained extracts and fractions were tested for their anti-biofilm growth activity against *C. albicans* using XTT method. Compounds of active subfractions were identified by LC-MS. The hexane extracts from leaves and stems were the most active against the fungus with IC_50_ at 500 and 250 µg/mL. Their bioguided fractionation led to 4 subfractions with IC_50_ between 62.5 and 125 µg/mL. Most of the components identified in active subfractions were fatty acids and terpenoïds.

## 1. Introduction

*Candida albicans* is a commensal species of the human digestive and genito-urinary tracts, which can become an opportunistic pathogen in immunocompromised patients and elderly people [1]. Its ability to form biofilm is responsible for a reduced susceptibility to most of the conventional antifungal agents, including especially the very commonly used azole agents [2,3]. Unfortunately, biofilms can develop on various substrates such as epithelia and medical devices including dentures and catheters as well [4,5,6]. For example, it has been shown that the minimal inhibitory concentration (MIC) of amphotericin B and azoles on sessile *C. albicans* cells were often ten time superior than MIC obtained on planktonic cells [2].

Currently only lipid formulations of amphotericin B and echinocandins such as caspofungin and micafungin are active against *C. albicans* biofilms [7]. However, some cases of resistance have been reported and new molecules active against these microbial fortresses are needed [8].

Biodiversity is a large source of compounds with interesting potential as therapeutic molecules. For example, red berries, consumed worldwide are recognized for their benefic effects on human health [9]. The fruit from the shrub *Rubus idaeus* L. belonging to the *Rosaceae* family contains numerous interesting compounds such as polyphenols and terpenoids [10]. It has been used in traditional medicine for ages to treat gastrointestinal disorders and it displays many pharmacological actions such as antioxidant, anti-inflammatory and antimicrobial activities [11,12]. Moreover, we recently reported that this raspberry fruit prevented *C. albicans* adhesion [13].

The fruit is the part of the plant with an interesting commercial value although stems and leaves are by-products and have been less studied. Yet, all parts have been used as traditional remedies for decades. For instance, leaves have been used to treat diarrhea, sore throat, menstrual pain, relieve morning sickness and ease labor [14] and shoots have been used by Eastern European population to treat common cold, fever and flu-like infections [15]. Both parts contain polyphenolic compounds, such as tannins, flavonoids and phenolic acids [14,15]. Some terpenoids and fatty acids were also reported in the leaves [16]. Concerning their pharmacological actions, both parts demonstrated antioxidant, antimicrobial and anti-cancer properties [11,15,17]. However, to our knowledge, no report has been made up to now on the anti-biofilm activity of leaves or stems of *R. idaeus* against bacteria or fungi.

In this context, we assessed the ability of extracts of these two parts of *R. idaeus* to inhibit the growth of *C. albicans* biofilms. Stems of two different maturation stages were studied to consider the variability of the chemical composition according to the shrub pruning period. A phytochemical investigation bioguided by anti-biofilm tests was performed on the most active extracts in order to determine the compounds responsible for the activity that could lead to new prophylactic treatments against biofilm-related candidiasis.

## 2. Results and Discussion

**Figure 1:**
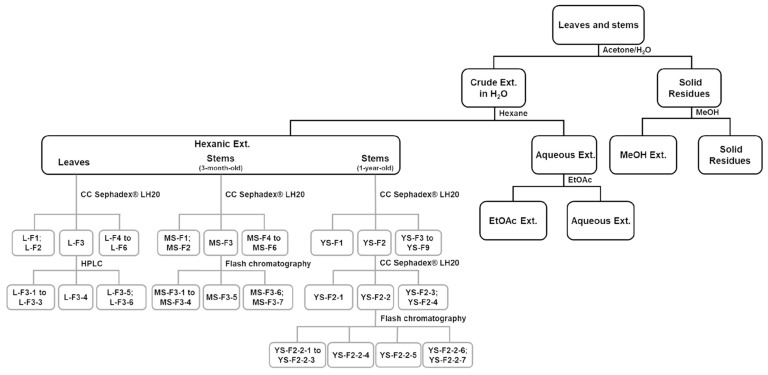
Summary of extraction and fractionation steps of *R. idaeus* leaves and stems.

Extractions were performed on *R. idaeus* leaves, 3-month-old and 1-year-old stems. The three hydroacetonic extracts were brought out by n-hexane and then EtOAc whereas the solid residues of the extractions were extracted by MeOH. Thus, twelve extracts were obtained (Figure 1).

Aqueous extracts were always obtained in largest amounts (10.2–16% yield) as well as EtOAc extracts to a lesser extent (1.3–3.2% yield) (Table 1) indicating the predominant presence of polar and moderately polar compounds in *R. idaeus* leaves and stems, which is coherent with the already known chemical composition of *R. idaeus*. Indeed, Gudej et al. (2004) and Krauze-Baranowska et al. (2014) previously mentioned that, in addition to polar primary metabolites such as sugars, *R. idaeus* leaves and stems contain a large amount of polyphenolic compounds [14,15]. According to Gudej et al. (2004), tannins can represent between 2.62% and 6.87% of the total dried weigh of the leaves [14]. It is known that these molecules mainly have high affinity for polar solvents such as water or EtOAc. Unfortunately, in our study, aqueous and EtOAc extracts were not able to significantly inhibit *C. albicans* biofilm growth (IC_50_ ≥ 1000 µg/mL). The MeOH extracts, obtained in lower amounts (0.5–1.8% yield) showed a weak anti-biofilm growth activity (IC_50_ ≥ 1000 µg/mL). The hexane extracts (0.4–1.1% yield) which contained more apolar compounds demonstrated the highest anti-biofilm growth activity (IC_50_ = 250–500 µg/mL, *p* < 0.05).

By comparing the yields of extracts from each part, we observed that leaves contained more compounds soluble in hexane and MeOH than stems (Table 1). The age of the stems appeared to influence their chemical composition: young stems were richer in polar compounds than older ones, which contained more compounds soluble in EtOAc. This observation agrees with the results obtained by Wang et al. (2000) who focused on red *R. idaeus* leaves and showed that their total phenolic content greatly diminished with age. For *R. idaeus* Autum Bliss cultivar leaves, the content decreased from 126.8 to 54.7 mg/g (dry matter; values expressed as milligrams of gallic acid equivalent) [18]. Moreover, another study performed by Shepherd et al. (1999) mentioned that the wax composition of leaves also varies following the stage of growth. Emerging leaves have lower levels of terpenes compared to older ones [19]. However, in our study, the age moderately influenced the biological activity: for instance, hexane and aqueous extracts of one-year stems were slightly more active than those obtained from 3-month-old stems (IC_50_ = 250 and 1000 µg/mL, respectively, versus 500 and 2000 µg/mL).

Due to their greater ability to inhibit the growth of *C. albicans* biofilms, the three hexane extracts were selected for a bioguided fractionation.

Two fractionation steps were performed from the leave hexane extract (Figure 1). A first step by Sephadex^®^ LH-20 generated six fractions from which L-F3 (40 mg) was the most active (IC_50_ = 50 µg/mL) (Table 2). This fraction was then fractionated by HPLC affording six subfractions, from which L-F3-4 (2 mg) was the most active (IC_50_ = 62.5 µg/mL).

The 3-month-old stem hexane extract was also fractionated. A Sephadex^®^ LH-20 step generated six fractions from which the most active was MS-F3 (40 mg, IC_50_ = 250 µg/mL) and then, the fractionation by flash chromatography of MS-F3 led to seven subfractions from which MS-F3-5 (2 mg) displayed the highest activity against biofilm growth (IC_50_ = 125 µg/mL).

Three steps were necessary to obtain two subfractions enriched in active compounds from 1-year-old stem hexane extract: two passages over Sephadex^®^ LH-20 generating first nine fractions including YS-F2 (295 mg) active at low concentration (IC_50_ = 100 µg/mL and then four subfractions obtained from YS-F2 including YS-F2-2 (186 mg) with the same activity (IC_50_ = 100 µg/mL). This last subfraction was subjected to flash chromatography which led to seven subfractions including the two enriched subfractions YS-F2-2-4 (16 mg) and YS-F2-2-5 (10 mg) showing IC_50_ = 62.5 and 125 µg/mL respectively.

**Figure 2:**
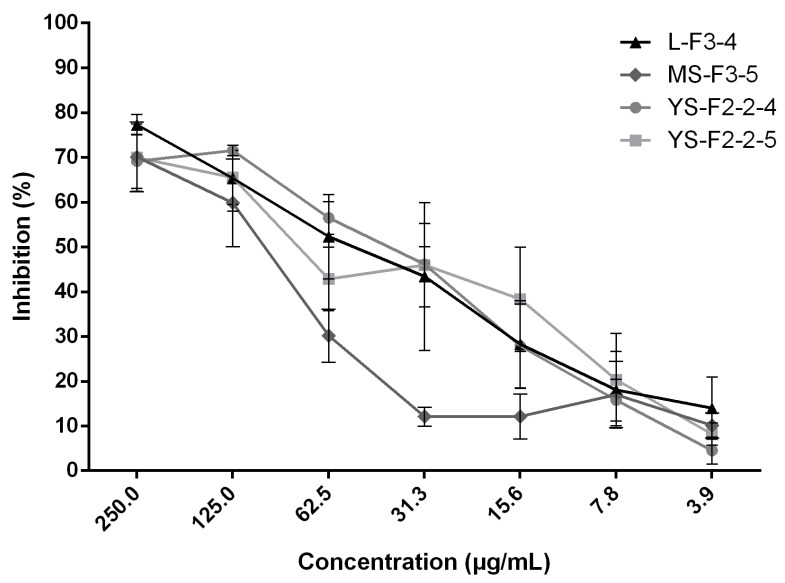
Anti-biofilm growth activity of the 4 subfractions L-F3-4, MS-F3-5, YS-F2-2-4 and YS-F2-2-5 enriched in active compounds. Results are expressed as mean of the inhibition percentages of *C. albicans* biofilm growth ± standard deviations depending on the concentrations of the subfractions. All experiments were performed in triplicate.

Overall, the fractionation steps led to four subfractions more active than the crude extracts (IC_50_ = 250–500 µg/mL for the extracts versus IC_50_ = 62.5–125 µg/mL for the enriched subfractions). These four final subfractions showed dose-dependent activities with the strongest biofilm growth inhibition of approximately 70% (250 µg/mL) (Figure 2).

**Figure 3:**
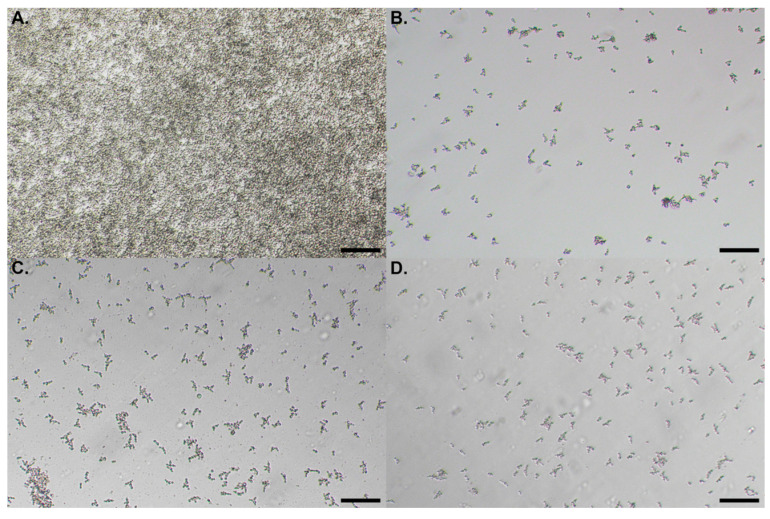
Microscopic observations of 24 h *C. albicans* biofilm non-treated (**A**) or treated with 2 mg/mL of hexane extracts from 1-year-old stems (**B**), 3-month-old stems (**C**) and leaves (**D**) of *R. idaeus* (10× objective, scale-bar represents 20 μm).

**Figure 4:**
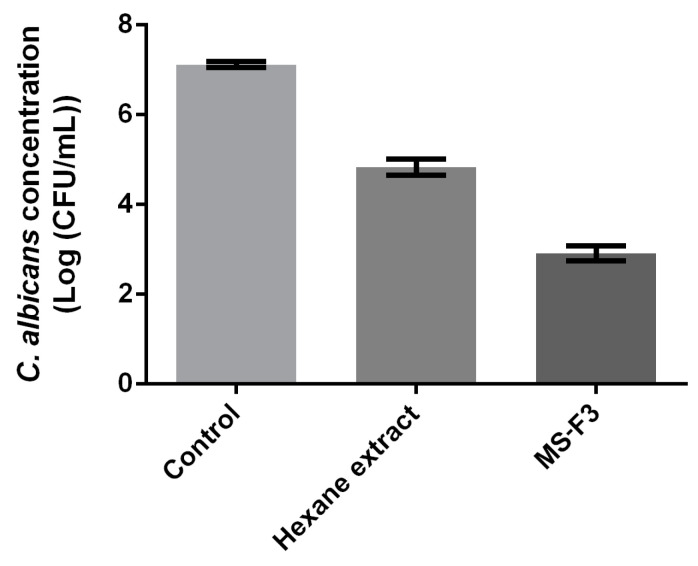
CFU counts after 24 h treatment of *C. albicans* cells with 1 mg/mL of hexane extract from 3-month-old stems, MS-F3 or without treatment.

During the bioguided fractionation, no false positive was detected by microscopic observations and/or by colony forming units (CFU) counting. Indeed, the decrease of absorbance values suggesting that the extracts, fractions or subfractions are active was always concomitant with a decreased amount of cells as shown by microscopic observation or CFU counts. Figure 3 illustrates the microscopic observations of 24 h *C. albicans* biofilm non-treated (A.) or treated with 2 mg/mL of hexane extracts from 1-year-old stems (B.), 3-month-old stems (C.) and leaves (D.) of *R. idaeus*, showing the weaker cell density on pictures B, C and D compared to A. Moreover, based on CFU results, a decrease in viability and cultivability was observed in the presence of the tested active compounds compared to non-treated cells, the difference of yeast concentrations reaching several logs. For example, a difference of two and four logs was observed on *C. albicans* concentration between the non-treated cells and those treated with 1 mg/mL of hexane extract from 3-month-old stems and MS-F3, respectively (Figure 4).

In order to determine the compounds responsible for the activity, the chemical compositions of the four active subfractions L-F3-4, MS-F3-5, YS-F2-2-4 and YS-F2-2-5 were analyzed by LC-MS in the negative ion mode. The main compounds were identified by comparison to literature data and by using mass database (NORMAN MassBank and Lipidbank (JCBL)). They would be mostly nonpolar compounds of lipid (fatty acids, phenolic lipid) and triterpenoid types that is in accordance with the fact that these compounds were obtained from apolar hexane extracts. Some polyphenols of tannins (p-galloyl-p-coumaroyl-p-cinnamoyl glucose), flavonoside (kaempferol-3-*O*-malonyl glucoside (Figure S5F)) and isoflavone (daidzein-8-C-glucoside (Figure S5E)) types were also observed in the subfractions L-F3-4, MS-F3-5 and YS-F2-2-4. One compound could not be identified which may suggest a not yet described compound. Some compounds can be found in two active subfractions such as 12,13-epoxy-9*Z*-octadecenoic acid (Figure S5C) and its hydroxylated derivative trihydroxy-octadecenoic acid (L-F3-4 and YS-F2-2-4) and 9-Oxo-10*E*,12*Z*-octadecadienoic acid (Figure S5B) (MS-F3-5 and YS-F2-2-5) (Table 3). Dimer forms were also observed in addition to the monomeric form such as 13*S*-hydroperoxy-9*Z*,11*E*-octadecadienoic acid dimer in MS-F3-5, 12,13-epoxy-9*Z*-octadecenoic acid dimer in YS-F2-2-4 and 9-Oxo-10*E*,12*Z*-octadecadienoic acid dimer in YS-F2-2-5.

Polyphenols, fatty acids and triterpenoids have previously been described in *R. idaeus* leaves [16,19] but to our knowledge, this is the first time that fatty acids and triterpenoids have been described in their stems.

More precisely, the compounds identified in these active subfractions are described to our knowledge for the first time in *R. idaeus* leaves and stems. Only ursolic acid-based triterpenoid was previously described in *R. idaeus* fruits as the aglycone of a major glycoside compound [21]. The other compounds were described in plants other than *R. idaeus* [22,24,25,26] or these are derivative compounds from these identified structures that have previously been described in *R. idaeus* or *Rubus* genus, strengthening attempts to identify these compounds [27,28,29,30,31]. 

The anti-biofilm growth effect against *C. albicans* observed during this study would be linked to one or several of these compounds, alone or in association with each other. To our knowledge, this anti-biofilm growth effect is described for the first time for all the identified compounds except for the phenolic lipid, anacardic acid. This compound is one of the 6-alkylated-2-hydroxybenzoic acids also called in their whole by the generic name “anacardic acid”. Anacardic acid inhibited some quorum-sensing related virulence factors such as pyocyanin and rhamnolipids production in *P. aeruginosa* [32]. Sajeevan et al. mentioned that *S. aureus* colonization and biofilm formation were reduced on anacardic acid-impregnated catheter tubes [33]. However, at our knowledge, its anti-*Candida* biofilm activity was described for the first time.

Yet, some of these identified compounds have previously shown activity against planktonic microbes. Thus 12,13-epoxy-9*Z*-octadecenoic acid previously showed an effect against planktonic *C. albicans* and some bacterial species such as *Bacillus subtilis* [26] or 13(*S*)-HPODE showed antifungal properties against several phytopathogenic species such as *Cladosporium herbarium*, *Alternaria brassicae* and *Leptosphaeria maculans* [34].

Furthermore, derivative compounds from these identified structures have previously shown antimicrobial and/or anti-biofilm activities such as ursolic acid which previously demonstrated anti-biofilm activity against bacteria strains [35] or linoleic acid which previously demonstrated antifungal activity against planktonic *C. albicans* yeasts [36] and anti-biofilm activity against the bacteria *Streptococcus mutans* [37]. Several extracts containing eicosadienoic acid (from which derive 15*S*-hydroperoxy-11*Z*,13*E*-eicosadienoic acid, also called 15(*S*)-HPEDE), showed antibacterial activity and inhibited *S. aureus* biofilm formation [38]. A publication by Rendeková et al. mentioned a good activity of an extract of *Cotinus coggygria* leaves rich in gallotannins against *S. aureus* biofilms [39]. Anti-biofilm and anti-adhesion effect of kaempferol against *S. aureus* was also described [40] and Freires et al. also mentioned the antifungal effect of a fraction containing this molecule against several species of *Candida* spp. [41]. Finally, the genin daidzein showed antimicrobial and anti-biofilm activities against soybean symbiont *Bradyrhizobium japonicum* [42] and inhibited also *E. coli* biofilm formation [43].

Some anti-biofilm mechanisms of the three categories of compounds identified in this study: lipids (fatty acids, phenolic lipid), triterpenoids and polyphenols, have been previously broached in the literature. It is known that adhesion, maturation or dispersion steps of biofilm life cycle can be targeted by these three categories of compounds. For example, free fatty acids, triterpenoids as gymnemic acids and polyphenols as resveratrol can influence the hyphal growth of *C. albicans*, which is a key feature during the biofilm formation, and/or suppress its germination in vitro [44,45,46,47]. Other compounds of these three classes can also impact the biofilm by some quorum quenching activities as it was previously shown with molecules such as *cis*-2-decenoic or *cis*-9-octadecenoic acids (lipids), ursolic acid (terpenoid) or kaempferol (polyphenol) [48,49,50].

Thus, it is now necessary to isolate pure compounds in order to exactly identify the molecule(s) responsible for the anti-biofilm activity and to understand their mechanism(s) of action, for example, by electronic microscopy observations, by studying the impact on the hyphal growth of *C. albicans* or by studying some quorum quenching activities.

## 3. Materials and Methods

### 3.1. General Experimental Procedures

Analytical TLC were carried out on precoated silica gel 60 F254 plates from Sigma-Aldrich (St. Louis, MO, USA). Methanol (MeOH)/CH_3_COOH/H_2_O (60:1:39) and butanol (BuOH)/CH_3_COOH/H_2_O (3:1:1) were used as mobile phase. Spots were detected under UV light (254 and 365 nm) before spraying with sulphuric vanillin or Liebermann–Burchard reagents.

Column chromatography was performed on Sephadex^®^ LH20 from Sigma-Aldrich. The mobile phases were first a mixture of H_2_O/MeOH (80:20 to 0:100, *v*/*v*) then H_2_O/acetone (30:70 to 0:100, *v*/*v*).

Flash chromatography was performed with a Puriflash^®^ 4250 from Interchim (Montluçon, France) equipped with a diode array detector and flash column (C-18, 30 µm, 12 g, Interchim). UV detection was monitored at 220 and 265 nm. The samples were solubilized in methanol or adsorbed on dicalite from Acros organics (Fair Lawn, NJ, USA) to perform a dry loading. The solvents of the mobile phase were water (solvent A) and a mix acetonitrile/water (90:10) (solvent B) with a gradient of 6% to 100% B in 35 min and then 15 min at 100% B at 5 mL/min.

Analytical HPLC was performed on a Dionex Ultimate 3000^®^ equipped with a diode array detector and the fractionation was performed on a Dionex HPLC system equipped with a P680 pump and a UV Ultimate 300^®^ series detector (Thermo Fisher Scientific, Waltham, MA, USA). The systems were fitted with a Dionex Acclaim^®^120, C18 (4.6 × 250 mm, 5 µm particle size, 120 Å) column, itself protected by a Phenomenex^®^ SecurityGuard (Torrance, CA, USA). UV detection was monitored at 220 and 265 nm. Samples were injected at 2.5 mg/mL in MeOH after centrifugation. The solvents of the mobile phase for analytical HPLC were water (solvent A) and a mix acetonitrile/water (90:10) (solvent B) with a gradient of 6% to 100% B in 35 min and then 10 min at 100% B at 0.8 mL/min. For the fractionation the gradient was changed to: 6% to 90% B in 7 min, 5 min at 90% B and then 10 min at 100% B.

A Waters system equipped with a time-of-flight XEVO™ G2 Q-TOF analyzer (Waters Corporation, Milford, MA, USA) and an ElectroSpray Ionization (ESI) source were used to carry out the mass spectrometry (MS) analyses. Samples were solubilized at 1 mg/mL in MeOH and then half-strength with H_2_O/acetonitrile (50/50—*v*/*v*) + 10 mM ammonium formate. A total of 7 µL of samples were eluted by H_2_O/acetonitrile (50/50 *v*/*v*) + 10 mM ammonium formate at 0.5 mL/min during 5 min. For LC-MS, the LC separation was conducted using a Dionex Acclaim^®^120, C18 (4.6 × 150 mm, 5 µm particle size, 120 Å, Thermo Fisher Scientific) column. The solvents of the mobile phase were water + 10 mM ammonium formate (solvent A) and acetonitrile/water (95:5) + 10 mM ammonium formate (solvent B) with a linear gradient of 40% to 100% B in 30 min and then 10 min at 100% B at 0.5 mL/min. A total of 10 µL of samples prepared in MeOH were injected. Data were obtained using the MS function in centroid mode, with a 5 V collision energy for MS analysis and a collision energy ramp of 5–40 V for MS/MS analysis. The source temperature was set to 120 °C. Negative ionization mode and acquiring data between 50 and 1500 *m*/*z* were applied to obtain the mass spectra. Data were analyzed using MassLynx software from Waters.

### 3.2. Plant Material

*R. idaeus* leaves, 3-month-old stems and 1-year-old stems (Polka cultivar) were collected in May 2016 (leaves and 3-month-old stems) and February 2015 (1-year-old stems), at Les vergers de Chézeau, Baille-Barrelle farmhouse, Roches-Prémarie-Andillé (France). A voucher specimen of each part was deposited at the Herbarium of the School of Pharmacy at the University of Poitiers (France) (registration numbers: RI L0516; RI S0516; RI S0215). The plant parts were air-dried at room temperature in the dark and pounded by a hammer mill for the stems and with mortar and pestle for the leaves.

### 3.3. Preparation of Extracts

Dried and powdered leaves, 3-month-old and 1-year-old stems (50 g, 50 g and 500 g, respectively) were macerated three times for 24 h in acetone/H_2_O 60:40 *v*/*v* solvent (1 L, 1 L and 12.5 L, respectively) with constant shaking at room temperature, protected from light. After pooling the obtained filtrates, acetone was evaporated under reduced pressure at 40 °C. The obtained aqueous crude extracts were then extracted three times by two solvents with increasing polarity: n-hexane and then ethyl acetate (EtOAc) to fractionate the compounds according to their polarity. The solid residues of extractions were finally macerated three times for 24 h in MeOH (400 mL, 400 mL, 5 L) with constant shaking at room temperature, protected from light and then filtrated. All obtained extracts (hexane, EtOAc, MeOH and H_2_O) were evaporated to dryness under reduced pressure at 40 °C. Thus, four extracts were obtained for each part of *R. idaeus* (total of 12 extracts) (Figure 1).

### 3.4. Fractionation of Active Extracts

Leaves: the hexane extract was subjected on passage over Sephadex^®^ LH-20 generating six fractions based on TLC and HPLC analysis. F3 (L-F3) was subjected to HPLC to afford 6 subfractions including L-F3-4.

Three-month-old stems: the hexane extract was subjected on passage over Sephadex^®^ LH-20 generating 6 fractions based on TLC and HPLC analysis. F3 (MS-F3) was subjected to flash chromatography on reverse phase generating seven subfractions including MS-F3-5.

One-year-old stems: the hexane extract was subjected on passage over Sephadex^®^ LH-20 generating nine fractions based on TLC and HPLC analysis. F2 (YS-F2) was fractionated by passage over Sephadex^®^ LH-20 generating four subfractions based on TLC and HPLC analysis. YS-F2-2 was subjected to flash chromatography on reverse phase generating seven subfractions including YS-F2-2-4 and YS-F2-2-5 (Figure 1).

### 3.5. Anti-Biofilm Growth Test

Stock solutions of extracts, fractions and subfractions were prepared at the required concentration in DMSO (100, 50, 20 or 10 mg/mL).

All assays were performed on *C. albicans* ATCC^®^ 28367^TM^ which was purchased from the American Type Culture Collection.

*C. albicans* was first grown on Sabouraud glucose with chloramphenicol (0.05 g/L) plates (SGC) (Sigma-Aldrich) for 24 h at 37 °C. Prior tests, the yeast was cultured overnight at 37 °C in Yeast Nitrogen Base medium from Sigma-Aldrich, supplemented with 5 g/L glucose (YNB-Glc). This culture was then centrifuged for 10 min at 2000× *g*. The pellet was washed by centrifugation (2000× *g*, 10 min) with PBS and finally suspended at final concentration of 4 × 10^7^ cells/mL in YNB-Glc.

Serial twofold dilutions of each stock solution of extracts, fractions or subfractions were prepared in YNB-Glc in untreated 96-well tissue culture polystyrene plates before adding the same volume per well of yeast culture. Some wells were reserved for non-treated yeasts (negative control) and yeasts treated by DMSO 2% (control of the solubilization solvent). Microplates were incubated for 24 h at 37 °C. Spent media and free-floating microorganisms were then removed by aspiration and wells were washed once with PBS (200 µL). The biofilm was then quantified using a previously described metabolic assay based on the reduction of a tetrazolium salt (XTT) [51]. Briefly, 100 µL of PBS and 50 µL of an extemporaneously prepared XTT-menadione mixture (4.35 mL of PBS, 600 µL of XTT solution (1 mg/mL of XTT in Ringer′s lactate from Baxter) and 60 μL of menadione solution (8.6 mg/mL in acetone) for a 96 well microplate) were added per well. Microplates were incubated for 3 h at 37 °C. Following incubation, absorbance of XTT formazan was measured at 492 nm (Sunrise™ absorbance reader from Tecan). Optical microscopy observations (IX51 inverted microscope from Olympus) were done before XTT addition for each test to prevent false positive signals. CFU were also counted for some samples. Thus, sessile cells from treated and untreated wells were removed from the microplates bottom by scraping and extensive rinsing. Obtained suspensions were sonicated during 10 min before being plated on SGC plates after adequate dilutions. After 24 h of incubation at 37 °C, CFU were counted.

All experiments were performed in triplicate with at least three replicate experiments. The inhibitory percentages and the concentration that inhibited 50% of the biofilm formation (IC_50_) were determined for each tested sample by constructing a dose-response curve and selecting the closest tested concentration value above or equal to 50% inhibition.

### 3.6. Statistical Analysis

Mann–Whitney test was applied to determine statistical significance of the differences between the groups. Differences were considered significant if *p* < 0.05.

## 4. Conclusions

Further investigations are needed to exactly identify the molecule(s) responsible for the anti-biofilm activity and to understand their mechanism(s) of action. To our knowledge, the thirteen compounds identified in the present report are described for the first time in *R. idaeus* leaves and stem. Furthermore, this work also highlights for the first time the potential of these red *R. idaeus* parts to prevent *C. albicans* biofilm formation. Finally, this study completes the available data concerning the chemical composition and pharmacological activities of *R. idaeus* leaves and stems. It shows that these parts, which are by-products of red *R. idaeus* fruits production, could be a source of new and innovative molecules to inhibit the formation of *C. albicans* biofilm.

## Figures and Tables

**Table 1:** Weight, yield and anti-biofilm growth activity against *C. albicans* of hexane, ethyl acetate, methanolic and aqueous extracts obtained from *R. idaeus* leaves, 3-month and 1-year old stems. Biological results are expressed as 50% inhibitory concentration (*p* < 0.05). All experiments were carried out in triplicate with at least three replicate experiments.

*R. idaeus*	Extracts	Weight (g)	Yield (%)	Anti-Biofilm Growth Activity IC_50_ (μg/mL)
Leaves	Hexane	0.56	1.1	500
	EtOAc	1.57	3.2	1000
	MeOH	0.88	1.8	>2000
	Aqueous	5.08	10.2	2000
3-month-old stems	Hexane	0.23	0.5	500
	EtOAc	0.62	1.3	2000
	MeOH	0.41	0.8	1000
	Aqueous	8.01	16	2000
1-year-old stems	Hexane	1.76	0.4	250
	EtOAc	13.87	2.8	>2000
	MeOH	2.65	0.5	>2000
	Aqueous	58.93	11.8	1000

**Table 2:** Weight and anti-biofilm growth activity against *C. albicans* of all fractions obtained from hexane extracts of *R. idaeus* leaves, 3-month and 1-year-old stems. Biological results are expressed as 50% inhibitory concentration (*p* < 0.05). All experiments were carried out in triplicate with at least three replicate experiments.

*R. idaeus*	Fractions and Subfractions	Weight (mg)	Anti-Biofilm Growth Activity IC_50_ (μg/mL)
Leaves	L-F1; L-F2	50–80	200
	L-F3	40	50
	L-F4 to L-F6	50–290	≥200
	L-F3-1 to L-F3-3	5–7	≥250
	L-F3-4	2	62.5
	L-F3-5; L-F3-6	0.5–4	≥250
3-month-old stems	MS-F1; MS-F2	25–40	≥1000
	MS-F3	40	250
	MS-F4 to MS-F6	3–20	≥1000
	MS-F3-1 to MS-F3-4	0.8–3.5	≥250
	MS-F3-5	2	125
	MS-F3-6; MS-F3-7	2–5.3	≥250
1-year-old stems	YS-F1	227	>400
	YS-F2	295	100
	YS-F3 to YS-F9	10–247	≥400
	YS-F2-1	50	200
	YS-F2-2	186	100
	YS-F2-3; YS-F2-4	15–38	> 400
	YS-F2-2-1 to YS-F2-2-3	6–28	≥250
	YS-F2-2-4	16	62.5
	YS-F2-2-5	10	125
	YS-F2-2-6; YS-F2-2-7	5–17	≥250

**Table 3:** LC-MS analysis of active subfractions L-F3-4, MS-F3-5, YS-F2-2-4 and YS-F2-2-5: tentative identification, retention time, molecular formula, molecular weight (Mw), *m*/*z* ratio.

Fraction	Tentative Identification	RT (min)	Formula	Mw	MS Data (*m*/*z*)	MS/MS Data (*m*/*z*)	Reference
L-F3-4	12,13-epoxy-9*Z*-octadecenoic acid	21.44	C_18_H_32_O_3_	296.23	295.18 [M − H]^−^	277.29; 259.27; 233.28; 195.18; 183.14; 171.13; 113.11	UT000014 (NORMAN MassBank) CID 5,356,421 (PubChem Database)
	trihydroxy-octadecenoic acid	20.88	C_18_H_34_O_5_	330.24	329.19 [M − H]^−^	293.30; 211.18; 171.14	[20]
	Ursolic acid based triterpenoid	22.67			517.26	455.46; 375.11	[21]
	p-galloyl-p-coumaroyl-p-cinnamoyl glucose	30.83	C_31_H_28_O_13_	608.15	607.39 [M − H]^−^	571.64; 293.30	[22]
MS-F3-5	9-Oxo-10*E*,12*Z*-octadecadienoic acid	21.49	C_18_H_30_O_3_	294.21	249.02 [M− CO^2^ − H]¯	185.04; 125.12	[23]
	13*S*-hydroperoxy-9*Z*,11*E*-octadecadienoic acid	24.10	C_18_H_32_O_4_	312.23	311.29 [M − H]^−^	293.30; 223.23; 181.16; 171.14; 155.14	UT000068 (NORMAN MassBank)
	Unidentified	6.27			345.27	309.30; 291.28; 281.06; 238.22; 209.17; 197.16; 171.14	
	kaempferol-3-*O*-malonyl glucoside	10.27	C_24_H_22_O_14_	534.42	533.49 [M − H]^−^	487.50, 447.20, 285.10	[24]
	13*S*-hydroperoxy-9*Z*,11*E*-octadecadienoic acid dimer	24.14	(C_18_H_32_O_4_)2	312.23	623.61 [2M − H]^−^	511.51; 329.33; 311.31; 293.27; 249.03	UT000068 (NORMAN MassBank)
YS-F2-2-4	12,13-epoxy-9*Z*-octadecenoic acid	21.54	C_18_H_32_O_3_	296.23	295.26 [M − H]^−^	277.29; 259.27; 233.28; 195.18; 183.14; 171.13; 113.11	UT000014 (NORMAN MassBank) CID 5,356,421 (PubChem Database)
	trihydroxy-octadecenoic acid	22.10	C_18_H_34_O_5_	330.24	329.28 [M − H]^−^	293.30; 211.18; 171.14	[20]
	Anacardic acid	22.11	C_22_H_30_O_3_	342.21	341.28 [M − H]^−^	323.28 295.30; 277.29	[23]
	Daidzein-8-C-glucoside	6.30	C_21_H_20_O_9_	416.11	415.33 [M − H]^−^	295.31	[20]
	12,13-epoxy-9*Z*-octadecenoic acid, dimer	21.56	(C_18_H_32_O_3_)2	296.23	591.56 [2M − H]^−^	545.48; 329.33; 277.29; 195.18; 171.14	UT000014 (NORMAN MassBank) CID 5,356,421 (PubChem Database)
YS-F2-2-5	9-Oxo-10*E*,12*Z*-octadecadienoic acid	21.49	C_18_H_30_O_3_	294.21	293.25 [M − H]^−^	197.18; 149.12; 125.11	[23]
	15*S*-hydroperoxy-11*Z*,13*E*-eicosadienoic acid	23.56	C_20_H_36_O_4_	340.50	339.27 [M − H]^−^	321.27; 307.27	DFA8147 Lipidbank (JCBL)
	9-Oxo-10*E*,12*Z*-octadecadienoic acid, dimer	21.50	(C_18_H_30_O_3_)2	294.21	587.53 [2M − H]^−^	293.29; 265.21; 249.02	[23]

## Supplementary Materials

The following are available online at https://www.mdpi.com/1424-8247/13/12/477/s1, Figure S1: Mass spectrum of active subfraction L-F3-4; Figure S2: Mass spectrum of active subfraction MS-F3-5; Figure S3: Mass spectrum of active subfraction YS-F2-2-4; Figure S4: Mass spectrum of active subfraction YS-F2-2-5; Figure S5: Chemical structures of some compounds identified in active subfractions: 15*S*-hydroperoxy-11*Z*,13*E*-eicosadienoic acid (A.), 9-Oxo-10*E*,12*Z*-octadecadienoic acid (B.), 12,13-epoxy-9*Z*-octadecenoic acid (C.), 13S-hydroperoxy-9Z,11*E*-octadecadienoic acid (D.), daidzein-8-C-glucoside (E.) and kaempferol-3-*O*-malonyl glucoside (F.)
