# Supplementary material for: Can Leaves and Stems of Rubus idaeus L. Handle Candida albicans Biofilms?
Source: Pharmaceuticals (Basel). 2020 Dec 18;13(12):477. doi: 10.3390/ph13120477 (PMC7766086; doi:10.3390/ph13120477)
Supplement: Supplementary file 1 [file pharmaceuticals-13-00477-s001.pdf]

Can Leaves and Stems of *Rubus idaeus* L. Handle *Candida albicans* Biofilms?

Clément Bernard, Camille Juin, Marine Vitry, Van Thanh Danh Le, Julien Verdon, Anne-Solène Toullec, Christine Imbert and Marion Girardot *

Laboratoire Ecologie et Biologie des Interactions, Université de Poitiers, UMR CNRS 7267, Poitiers F-86073, France.

* Correspondence: marion.girardot@univ-poitiers.fr; Tel.: +33-549-453-700

Abstract: *Candida albicans* is an opportunistic pathogen involved in many infections, especially linked to implanted medical devices. Its ability to form biofilms complicates the treatment of these infections as few molecules are active against sessile *C. albicans*. The aim of this study was to evaluate the potential of leaves, three-month-old and one-year-old stems of *Rubus idaeus* L. against *C. albicans* biofilm growth. Extractions with a polarity gradient were carried out on hydroacetonic extracts and followed by fractionation steps. The obtained extracts and fractions were tested for their anti-biofilm growth activity against *C. albicans* using XTT method. Compounds of active subfractions were identified by LC-MS. The hexane extracts from leaves and stems were the most active against the fungus with IC_{50} at 500 and 250 μg/mL. Their bioguided fractionation led to 4 subfractions with IC_{50} between 62.5 and 125 μg/mL. Most of the components identified in active subfractions were fatty acids and terpenoïds.

Keywords: *Rubus idaeus* L.; raspberry; *Rosaceae*; *Candida albicans*; anti-biofilm; bioguided fractionation; stems and leaves

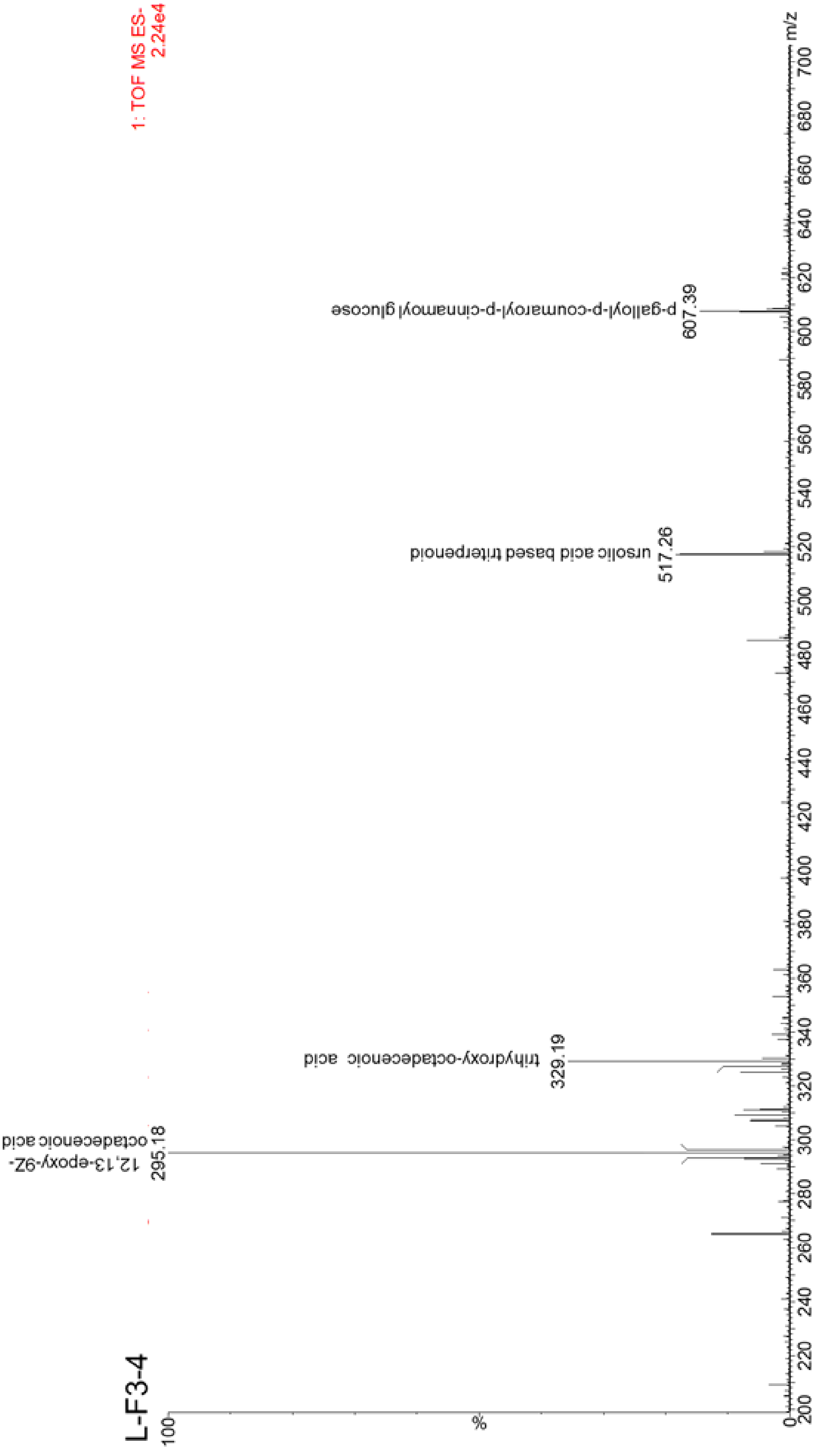

Figure S1. Mass spectrum of active subfraction L-F3-4

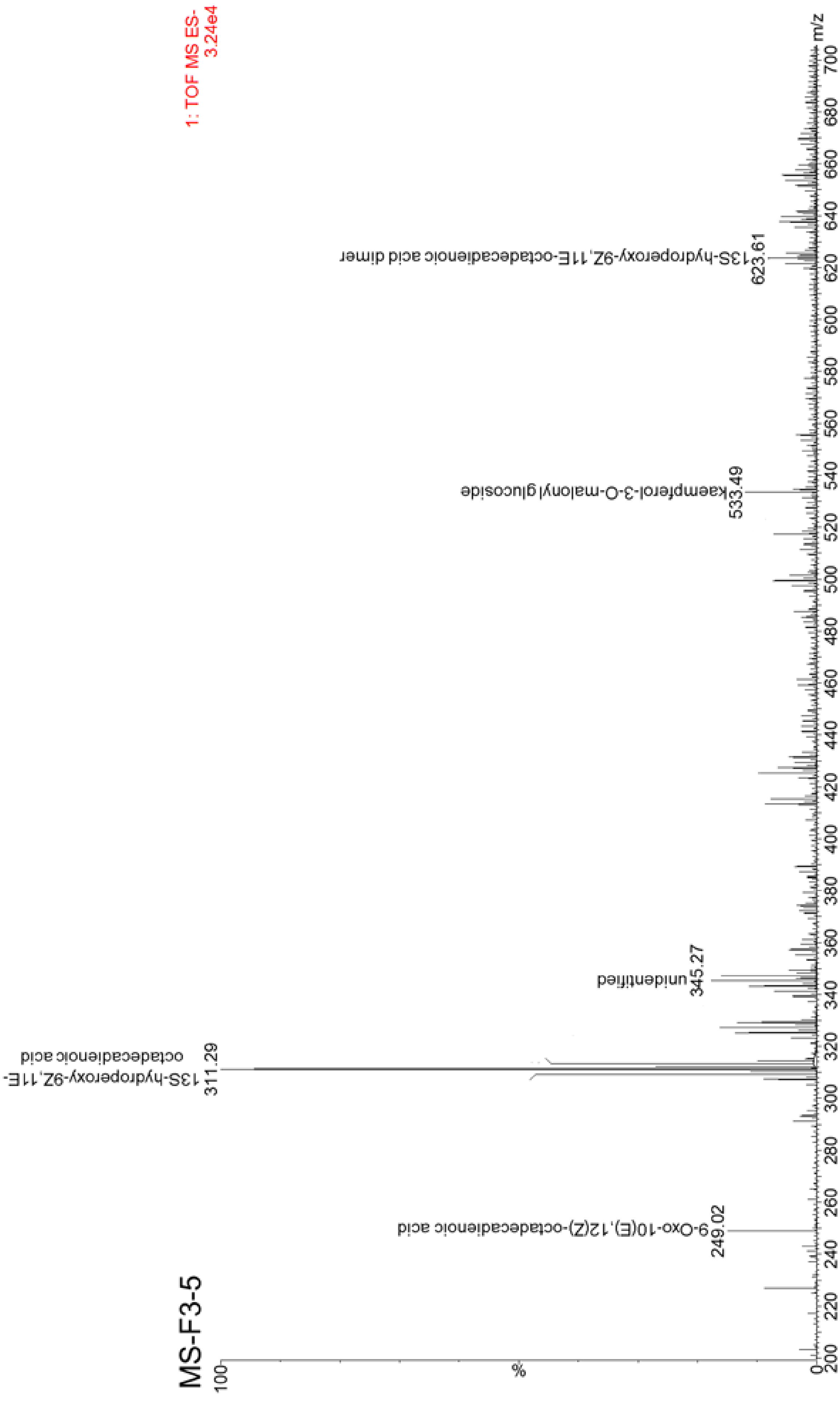

Figure S2. Mass spectrum of active subfraction MS-F3-5

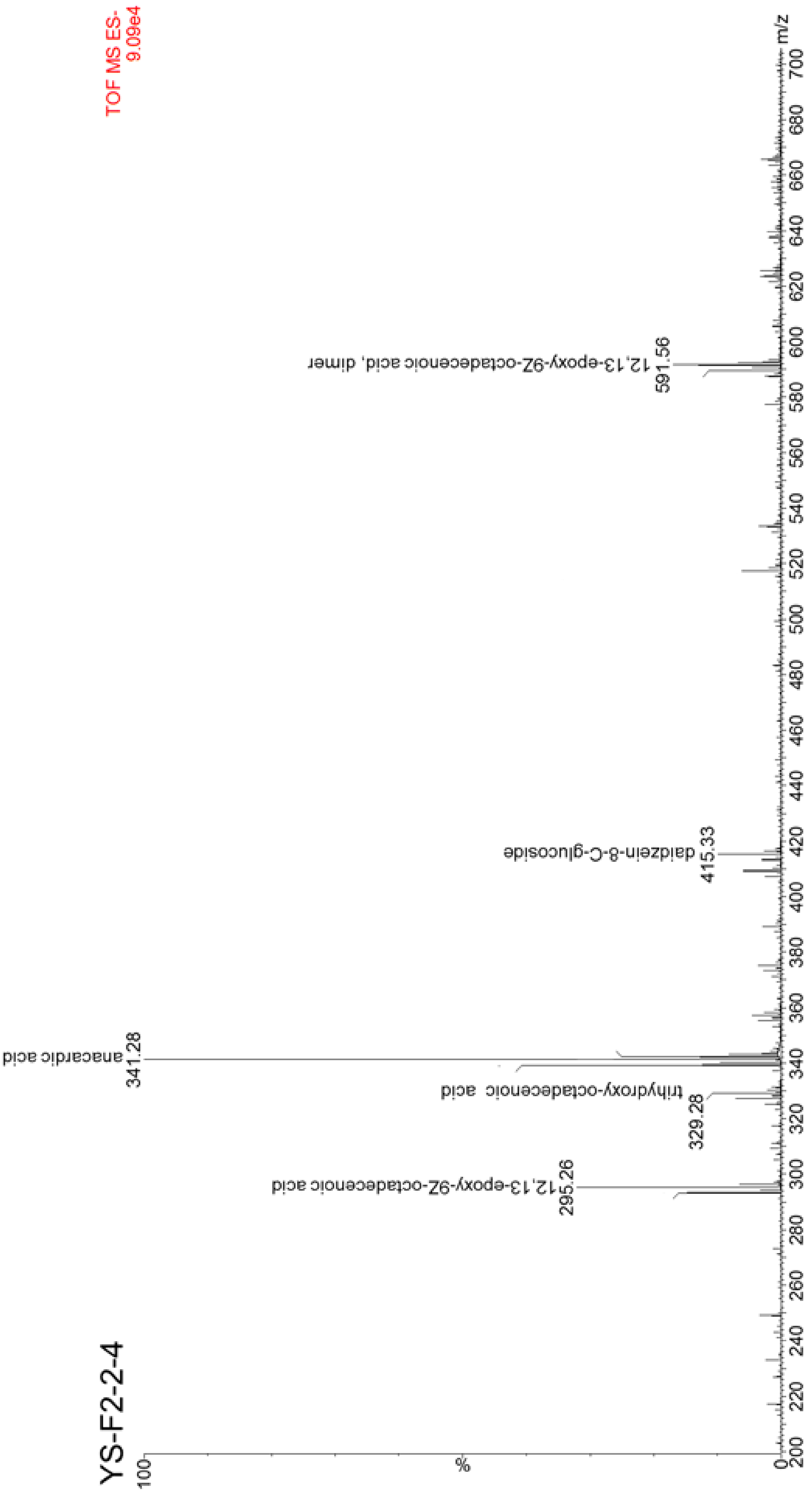

Figure S3. Mass spectrum of active subfraction YS-F2-2-4

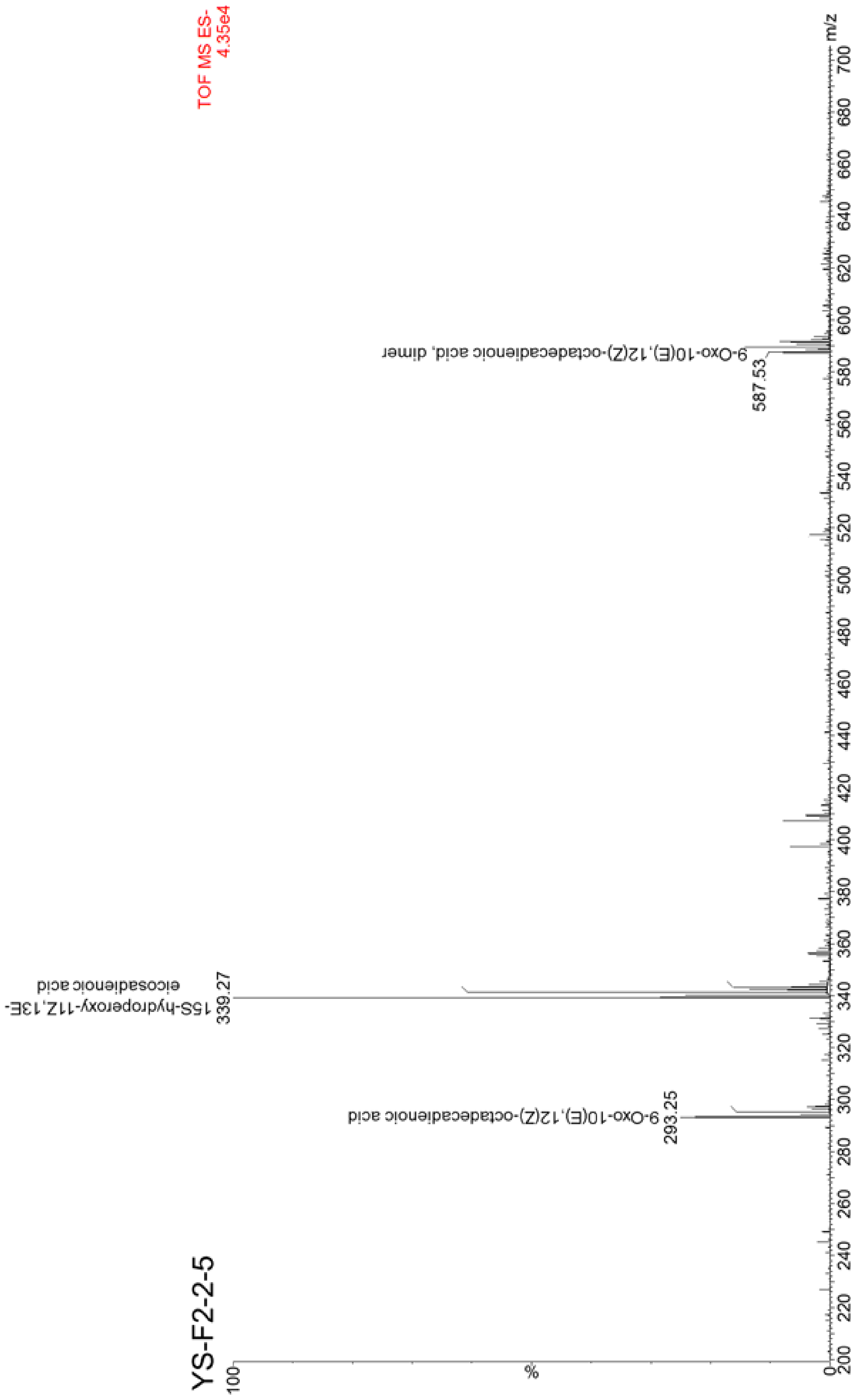

Figure S4. Mass spectrum of active subfraction YS-F2-2-5

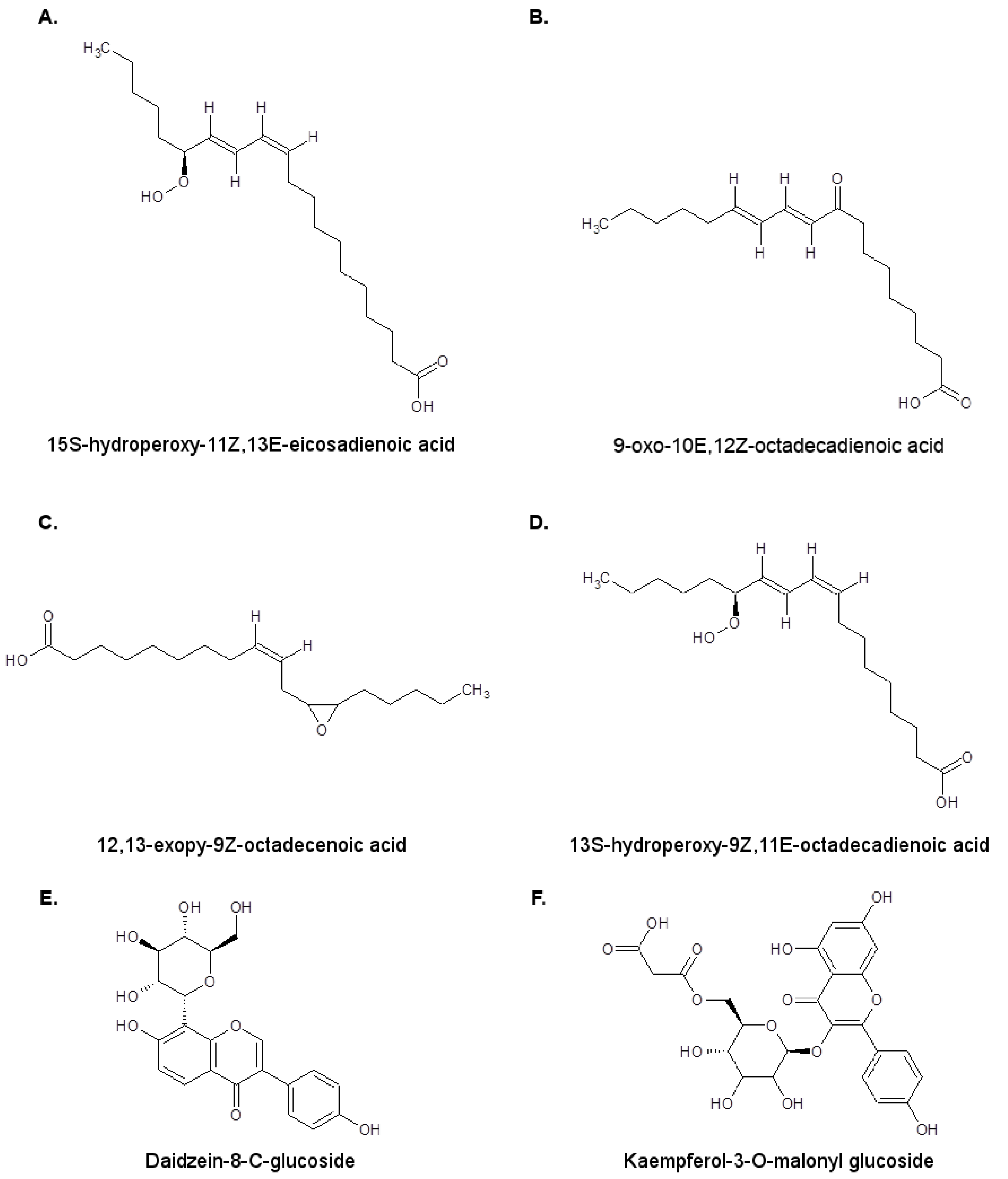

Figure S5. Chemical structures of some compounds identified in active subfractions: 15*S*-hydroperoxy-11*Z*,13*E*-eicosadienoic acid (**A.**), 9-Oxo-10*E*,12*Z*-octadecadienoic acid (**B.**), 12,13-epoxy-9*Z*-octadecenoic acid (**C.**),13*S*-hydroperoxy-9*Z*,11*E*-octadecadienoic acid (**D.**), daidzein-8-C-glucoside (**E.**) and kaempferol-3-O-malonyl glucoside (**F.**)
